# LEFT VENTRICULAR REMODELING PROCEEDS FROM YOUNG ADULTHOOD INTO MIDLIFE IN INTRAUTERINE GROWTH RESTRICTION BABOONS

**DOI:** 10.1093/geroni/igz038.396

**Published:** 2019-11-08

**Authors:** Geoffrey D Clarke, Hillary Huber, Cun Li, Anderson Kuo, Peter Nathanielsz

**Affiliations:** 1 UT Health San Antonio, San Antonio, Texas, United States; 2 University of Wyoming, Laramie, Wyoming, United States; 3 UNIVERSITY OF WYOMING, Laramie, Wyoming, United States; 4 Brigham and Women's Hospital, Boston, Massachusetts, United States

## Abstract

Previous cross-sectional studies have shown young adult baboons (~5-6 y.o.), subjected to intrauterine growth restriction (IUGR) by maternal calorie restriction during pregnancy and lactation, exhibit ventricular remodeling with mildly impaired heart function relative to age/sex-matched controls (CTL). METHODS: In this longitudinal study cardiac MRI was performed on male IUGR baboons (n=7). A 3 Tesla, Siemens TIM Trio MRI system was used with phase-array coils with parallel imaging acquisition and breath-holding during the scan. Studies of IUGR animals occurred at 4.7 + 0.1 yr. intervals; the first scan (scan1) at 5.8 + 1.2 y (human equivalent - HE ~24 years) and the second (scan2) at 10.4 + 1.2 yr (HE~40 y). Scans on the CTL animals (N=4) occurred at 5.3 + 1.4 years and 10 + 1.4 years. RESULTS: Change in body weight over 4.7 years was less in the IUGR group (Δwt=6.3 + 6.1 kg) than in the control group (Δwt =11.5 + 8.2 kg). Left ventricular (LV) ejection fraction (EF) was significantly greater in IUGR animals for scan2 (+10.7%, p=0.03) but not in normal controls (+1.8%, p=0.75). Stroke volume and end-diastolic LV volume were normalized to body surface area (BSA). SV/BSA (17.6 + 4.9, 31.5 + 12.3 mL/sq.m; p=0.016) and EDV/BSA (47.3 + 13.6, 64.5 + 18.8 mL/sq.m; p=0.045) were also significantly increased in IUGR animals but not controls. In IUGR subjects, Δweight was significantly and positively correlated with ΔEF (r=0.86, p=0.01). CONCLUSIONS: In IUGR, but not in CTL baboons, cardiac function adaptations continue into midlife and are related to increases in body weight with aging. We conclude that IUGR programs cardiovascular function and that programmed changes continue into midlife.

